# Climate‐Dependent Heat‐Triggered Opening Mechanism of *Banksia* Seed Pods

**DOI:** 10.1002/advs.201700572

**Published:** 2017-12-13

**Authors:** Jessica C. Huss, Vanessa Schoeppler, David J. Merritt, Christine Best, Eric Maire, Jérôme Adrien, Oliver Spaeker, Nils Janssen, Johannes Gladisch, Notburga Gierlinger, Ben P. Miller, Peter Fratzl, Michaela Eder

**Affiliations:** ^1^ Department of Biomaterials Max‐Planck Institute of Colloids and Interfaces Research Campus Golm 14424 Potsdam Germany; ^2^ BCUBE‐Center for Molecular Bioengineering Technische Universität Dresden Dresden 01307 Germany; ^3^ Kings Park and Botanic Garden Kings Park WA 6005 Australia; ^4^ School of Biological Sciences The University of Western Australia Crawley WA 6009 Australia; ^5^ INSA‐Lyon MATEIS CNRS UMR5510 University of Lyon F‐69621 Villeurbanne France; ^6^ RISE ICT/Acreo Bredgatan 33 SE‐60221 Norrköping Sweden; ^7^ Department of Nanobiotechnology University of Natural Resources and Life Science (BOKU) Muthgasse 11/II 1190 Vienna Austria

**Keywords:** banksia, dimensional stability, fire, triggered follicle opening

## Abstract

Heat‐triggered fruit opening and delayed release of mature seeds are widespread among plants in fire‐prone ecosystems. Here, the material characteristics of the seed‐containing follicles of *Banksia attenuata* (Proteaceae), which open in response to heat frequently caused by fire, are investigated. Material analysis reveals that long‐term dimensional stability and opening temperatures of follicles collected across an environmental gradient increase as habitats become drier, hotter, and more fire prone. A gradual increase in the biaxial curvature of the hygroscopic valves provides the follicles in the driest region with the highest flexural rigidity. The irreversible deformation of the valves for opening is enabled via a temperature‐dependent reduction of the elastic modulus of the innermost tissue layer, which then allows releasing the stresses previously generated by shrinkage of the fiber bundles in the adjacent layer during follicle drying. These findings illustrate the level of sophistication by which this species optimizes its fruit opening mechanism over a large distribution range with varying environmental conditions, and may not only have great relevance for developing biomimetic actuators, but also for elucidating the species' capacity to cope with climatic changes.

## 1 Introduction

**Figure 1:**
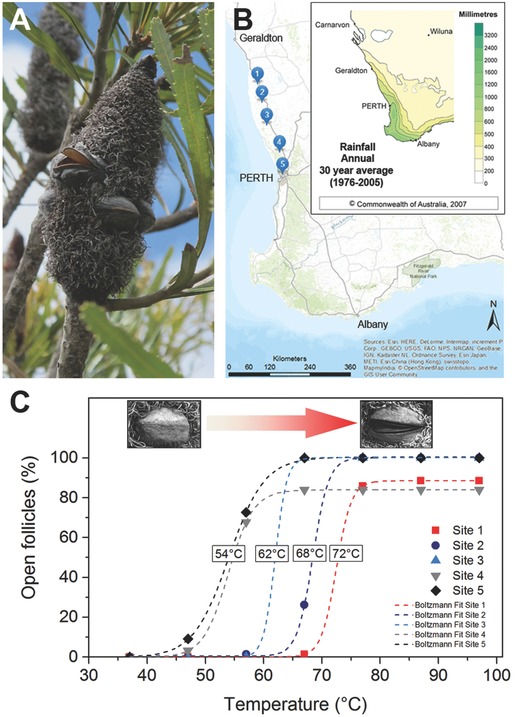
Characteristics of *B. attenuata* infructescences. A) Morphology of a mature infructescence in the field with open and closed follicles. B) Location of the five sampling sites along a climatic gradient between Geraldton and Perth (Western Australia). Inset map: annual rainfall distribution in the southwest of Western Australia. Adapted with kind permission of the Australian Bureau of Meteorology, 2007, Commonwealth of Australia. (http://www.bom.gov.au/jsp/ncc/climate_averages/decadal‐rainfall/index.jsp?maptype=6&period=7605&product=totals#maps; License:CC BY3.0AU, https://creativecommons.org/licenses/by/3.0/au/). C) Opening of initially closed follicles from different collection sites subjected to gradual heating with an increment of temperature every 24 h (temperatures at 50% opening indicated). Photos in diagram: closed state (left); open follicle after heating (right).

Plants are continuously exposed to environmental factors with high spatial and temporal fluctuations, e.g., light intensity, temperature, or humidity. In order to grow, survive, and reproduce in a highly variable environment, they use active (metabolic) and passive (nonmetabolic) mechanisms. Living cells and tissues are characterized by active metabolic processes that are involved in sensing and responding to environmental stimuli through sensory receptors and electric signals/action potentials.^1^ Specific metabolic pathways can even enable rapid movements of individual plant organs, as in the trap closure of the Venus flytrap.^2^ Passive mechanisms, on the other hand, are solely based on the physical interaction of dead plant material and its environment; realized by means of finely tuned tissue properties.^3^, ^4^, ^5^ Some structural features of plant cells, such as the orientation of the cellulose fibrils in the cell wall, can be controlled during their development and remain functional after cell death.^6^ In many plants, the physical interactions of dead, yet highly functional tissues with their surrounding environment underlie important processes; including seed release. Prominent examples are pine cones, in which bending of the cone scales occurs upon drying, leading to opening and seed release;^7^, ^8^ and the seed capsules of ice plants, which unfold via a hydration‐powered swelling movement.^9^ Besides purely humidity‐driven mechanisms, another fine‐tuned system for environmentally triggered seed release has evolved in the Australian plant genus *Banksia*, in which the seeds are commonly released from woody seed pods (more precisely “follicles”) (**Figure** **1**A) after exposure to fire.^10^, ^11^, ^12^ The genus *Banksia* is a member of the Gondwanan plant family Proteaceae,^13^ in which the trait of serotiny (delayed release of mature seeds) has been dated back to the Paleocene.^10^ Serotiny is a mechanism that provides seed protection during seed bank accumulation in the plant canopy and triggered mass release of seeds into conditions suitable for seedling establishment.^14^, ^15^ In order to maintain seed viability and provide for effective seed dispersal, which can occur several years after seed maturation,^12^ the long‐term integrity of the follicle structure and its functionality are essential. The follicles are exposed to tough environmental challenges, such as UV radiation, microbial attack, mechanical impact by bird predation, high ambient summer temperatures, and fire.^16^ It is unknown how the follicles are built in order to accomplish these functions. Moreover, it is not understood how the reported variations in the degree of serotiny^12^, ^15^ are reflected in follicle composition or opening temperature.

Here, we address these questions by reporting on the material properties and opening mechanism of follicles of *Banksia attenuata* R.Br. For this study, sample cones were sourced from plants across a climatic gradient of temperature and rainfall, in southwest Australia, where the species is endemic.^17^ Across this gradient, the species displays considerable plasticity in the degree of serotiny as habitat climate and fire regimes change.^15^ In regions with higher annual rainfall, *B. attenuata* spontaneously releases a higher number of seeds after maturity when compared to drier, more fire‐prone regions, where it tends to retain the majority of its mature seeds for longer time periods.^15^

Our study presents the temperature requirements to induce opening of *B. attenuata* follicles, together with a morphological and functional characterization of the follicles. We reveal intraspecific variation in the follicle opening temperature aligned to the climatic gradient, along with a previously unidentified mechanism for follicle opening. Previous research has proposed that resins with different melting points act as adhesives in the junction zone, and in this way account for the fire‐dependent follicle opening of some species.^12^ In contrast to this hypothesis, we experimentally show for *B. attenuata* that changes in the valve geometry, in combination with a temperature‐dependent decrease in the elastic modulus of the innermost tissue layer, is responsible for the opening gradient and the observed variation in serotiny.^15^

## 2 Results

### 2.1 Required Temperatures for Initial Follicle Opening

The initially closed follicles of *B. attenuata* cones (Figure 1A) collected from five locations along a 350 km latitudinal and climatic gradient (Figure 1B) commenced opening at temperatures ranging from *T* ≥ 47 °C at the southernmost (cooler/wetter) to *T* ≥ 67 °C at the northernmost (warmer/drier) site (Table S1, Supporting Information). Strikingly, the temperature threshold for initiating follicle opening was found to rise steadily as sampling sites progressed north (Figure 1C). Thus, the opening behavior follows the climatic gradient, which is characterized by strongly decreasing annual rainfall and increasing mean maximum temperatures in summer (Table S2, Supporting Information, and Figure 1B). From the reference follicles stored at 15 °C and 15% relative humidity (RH), only 1–2% of initially closed follicles from sites 2, 3, and 4 opened.

### 2.2 Follicle Organization

**Figure 2:**
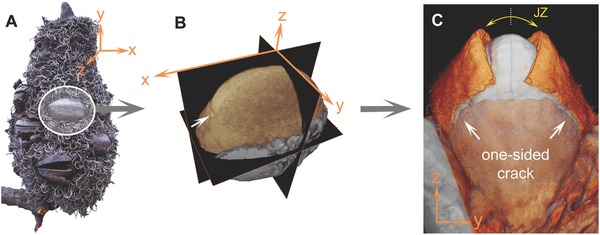
3D orientation and states of *B. attenuata* follicles. A) Spatial orientation of a follicle on the mature fruit cone. B) Directions of the three cutting planes. C) Overlay of two µCT scans of one follicle showing closed (gray) and open (colored) state with the one‐sided lateral crack after valve deformation and junction zone (JZ) separation.

The living plants form a seed bank in the canopy, with numerous infructescences (Figure 1A) that are metabolically inactive in the mature state, but can remain closed on the plant for several years.^12^, ^15^ The follicles are prominent features of the infructescences (**Figure** **2**A,B) and are tightly attached to the rachis, and surrounded by dried floral remnants. Upon follicle opening (Figure 2C), the region connecting the two follicle valves (hereafter termed “junction zone,” JZ), is exposed. The valve movement is accompanied by the formation of a lateral crack (Figure 2C; hereafter termed “fracture zone,” FZ), which always forms on the shorter side of the asymmetric follicles of *B. attenuata* (Figure 2B,C white arrows).

**Figure 3:**
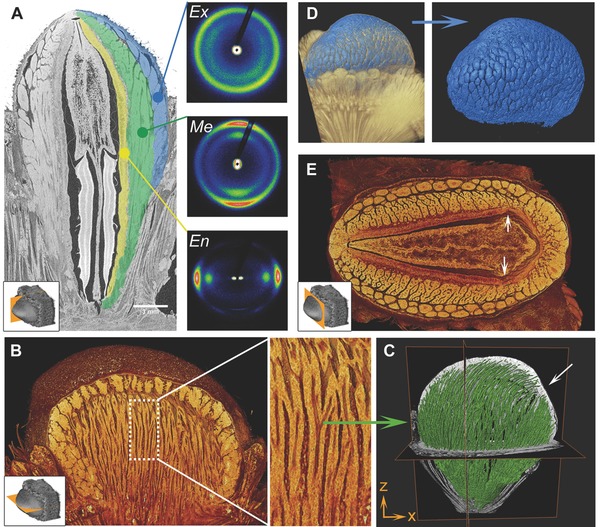
Internal structure and organization of the follicle tissue. A) Longitudinal section of a µCT scan with colored follicle valve showing endocarp in yellow, mesocarp in green, and exocarp in blue. The two seeds inside the follicle are held in place by a central separator plate. Insets: X‐ray scattering images for exocarp (Ex), mesocarp (Me), and endocarp (En) fibrous tissue measured on longitudinal sections. B) Transverse µCT section revealing the branching of mesocarp fiber bundles (zoom) and indicating the dark regions in each fiber bundle used for C) tracking of the mesocarp fiber bundles in the whole follicle (segmentation in green); arrow highlights the location of the lateral crack development. D) The segmented exocarp platelets (blue) are only found on the exposed follicle part and do not reach all the way into the rachis. E) µCT cross‐section revealing follicle asymmetry. The crack always develops on the shorter side of the follicle, where the thickness of the endo‐ and mesocarp are clearly reduced (arrows in (E) correspond to the one in (C)).

Botanically, *Banksia* follicles are woody fruit walls (pericarps) that are typically classified into three distinct tissue layers in each valve: the exo‐, meso‐, and endocarp (**Figure** **3**A; blue, green, and yellow colored areas). Each layer includes different proportions of parenchymatic and fibrous (sclerenchymatic) tissue; the two main tissue types of the valves (fibrous tissue appears bright, parenchymatic tissue dark in the microcomputed tomography (µCT) images). The comparably thin endocarp layer (Figure 3A, yellow) mainly contains fibrous tissue with the fibers predominantly oriented along the longitudinal axis of the follicles. The thicker mesocarp (Figure 3A, green) is composed of massive branching fiber bundles that are embedded in parenchymatic tissue. Figure 3B,C shows that the fiber bundles are branched, and mainly oriented along the longitudinal axis of the valves. The degree of branching increases from the follicle attachment (at the base) toward the JZ (at the tip). To resolve the tissue organization in the exocarp (Figure 3A, blue), fibrous tissue was segmented; revealing that it forms plate‐like agglomerates with parenchymatic tissue in‐between (Figure 3D). The cross‐section through the area of the FZ in Figure 3E (section planes indicated by icons) shows that the endo‐ and mesocarp have a reduced thickness in this region and that the follicle geometry is asymmetric.

Wide angle X‐ray scattering images (Figure 3A, insets) reveal no distinct orientation of cellulose fibrils in the exocarp. In contrast to this, the mesocarp fibers show a cellulose microfibril orientation that is mainly transverse to the fiber axis, whereas in the endocarp, the orientation of the cellulose microfibrils is rotated by about 90°. In analogy with other systems studied in great detail, such as wheat awns or pine cones,^5^, ^8^ this arrangement of microfibril angles (MFAs) implies that meso‐ and endocarp form a bilayer system, in which the mesocarp reacts to changes in moisture content by swelling and shrinking mainly along the fiber axis, whereas the endocarp remains dimensionally more stable in this direction. For those reasons, drying will induce the tendency of this bilayer to bend. The bending movement is, however, restricted by the endocarp, so that a considerable residual stress is likely to build up in the closed seed pods.

### 2.3 Initiation of Follicle Opening

**Figure 4:**
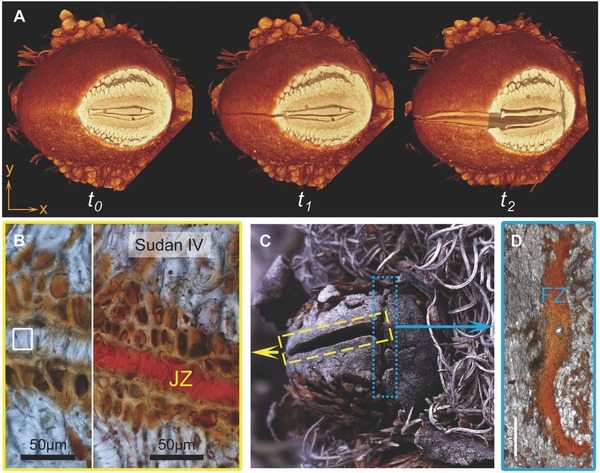
Time‐resolved opening and composition of specialized follicle tissue. A) µCT images taken of one follicle at different time points during heating. *t*_0_: before heating (FZ intact and JZ closed); *t*_1_: after some minutes of heat exposure (FZ intact, whereas JZ is already open); *t*_2_: after a longer period of heating (FZ broken, JZ open). B) Light micrograph of an unstained (left) and Sudan VI stained (right) junction zone (JZ) tissue section; indicating the presence of lipids in the JZ in bright red. C) Slightly open follicle with JZ and the one‐sided fracture zone (FZ) indicated. D) Unstained FZ tissue section under the light microscope (scale bar = 500 µm). The red coloration originates from the presence of condensed tannins in the tissue.

In order to understand the opening process in closer detail, fast µCT scans were acquired at different time points during follicle heating. The µCT data (**Figure** **4**A) shows that the JZ starts to separate first (*t*_1_), followed by the formation of a crack in the parenchymatic tissue of the FZ (*t*_2_). As a starting point, these results suggest that the temperature sensitive component(s) relevant for opening might possibly be found in the JZ and/or in the FZ. Therefore, tissues in these two areas were further investigated. The JZ is characterized by a complex 3D structure, consisting of interdigitating tooth‐like structures in a lipid‐rich substance (positive Sudan IV reaction in Figure 4B). On the exposed part of the follicle, the JZ merges into the FZ and runs perpendicular to it (Figure 4C). In contrast to the JZ, the FZ is characterized by tannin‐rich parenchymatic tissue (Figure 4D). In the following two sections, both zones are analyzed with regard to their chemical and thermal properties.

### 2.4 Junction Zone and Melting Temperature of the Wax

The opening process of the follicles involves a tissue separation in the junction zone of the two valves (Figures 2C and 4B,C). It has previously been suggested for three other *Banksia* species that a resinous bonding agent is present in this zone, which is expected to trigger follicle opening by melting in response to heat and releasing the stresses generated between the two valves.^12^ Therefore, a strong mechanical function has been attributed to the resinous substance. In our study however, we find that this concept does not account for the opening mechanism of *B. attenuata* follicles at least.

**Figure 5:**
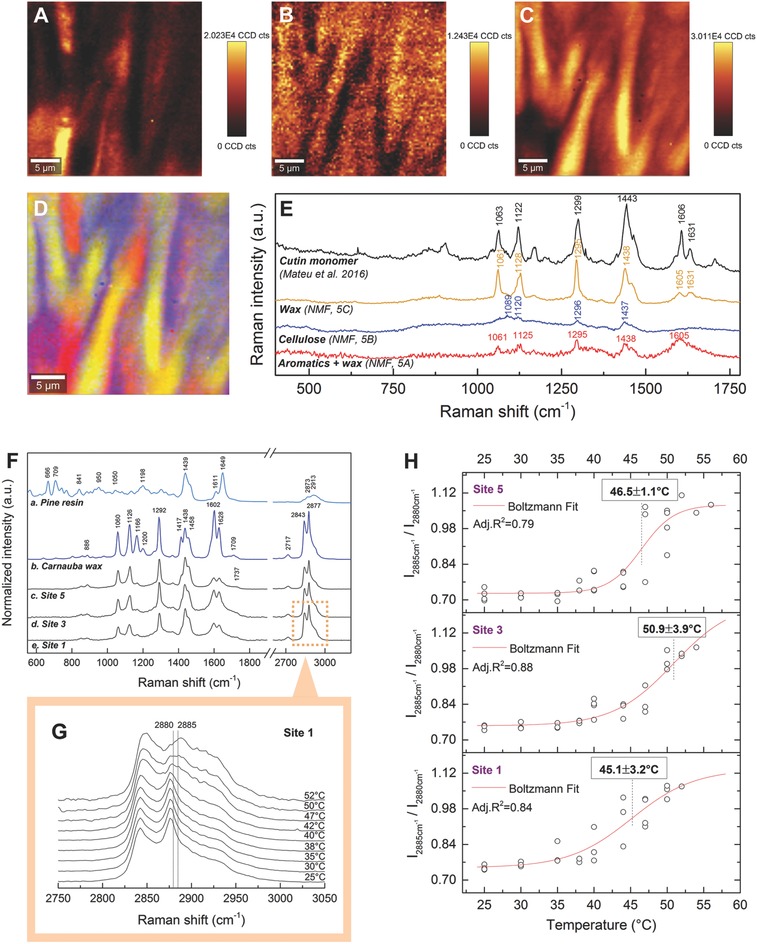
Physicochemical analysis of the junction zone. Chemical mapping (in situ Raman images obtained via non‐negative matrix factorization (NMF) analysis) of an area in the JZ (site 1, box in Figure 4B); indicating the presence of A) aromatic compounds/wax along with B) cellulose in the interdigitating tooth‐like structures and C) waxes (cutin) in between. D) Overlay of (A) (red), (B) (blue), and (C) (yellow). E) Spectra of cutin and the compounds in the JZ obtained via NMF. F) Raman spectra of two reference standards (*P. banksiana* resin and Carnauba wax) and of the JZs of *B. attenuata* follicles from three different sampling sites (all are in situ measurements at room temperature). Nonindicated peak positions in spectra (c–e) correspond to the ones in (b). G) Spectral features of the C—H stretching region at different temperatures. Relevant band positions are indicated. H) Melting behavior of the waxes present in the JZs of follicles from sites 5, 3, and 1 (inflection points indicated with ± SE). Data points represent the intensity ratio of the band at 2885 cm^−1^ relative to the one at 2880 cm^−1^.

Chemical mapping of a region in the JZ (white box in Figure 4B) by in situ Raman spectroscopy shows that the tooth‐like structures contain aromatic compounds associated with wax (**Figure** **5**A) and cellulose (Figure 5B). In between these structures, a 2–4 µm thick layer of wax is present (Figure 5C,D; corresponding spectra in Figure 5E), which shows identical peaks to the pure cutin monomer (Figure 5E). The identification of waxes/cutin was performed based on reference Raman spectra and vibrational band assignments according to the literature.^18^, ^19^, ^20^, ^21^ The Raman spectra of *B. attenuata* follicle waxes from three different sampling sites across the clinal range (Figure 5F) exhibit typical bands of long‐chain hydrocarbons, which can be detected due to the presence of several bands arising from C—H vibrational modes. These include the ν(CH_2_) asymmetric stretching band centered near 2877 cm^−1^, the ν(CH_2_) symmetric stretching band around 2843 cm^−1^, the ν(CH_3_—CH) band around 2717 cm^−1^, and a sequence of bands near 1458, 1438, 1417, 1370, and 1292 cm^−1^, all assigned to CH_2_ twisting, wagging and rocking. These bands are complemented by the carbon skeletal stretching bands ν(C—C) centered near 1166 and 1126 cm^−1^. The presence of esters can be identified based on the two stretching modes ν(C=O) and ν(C—O) located near 1737 (shifted to 1709 cm^−1^ in carnauba wax) and 1060 cm^−1^, respectively. In carnauba wax, the hydrocarbons are part of several esterified organic acids,^19^ and this is also evident in the lipids in the junction zone of *B. attenuata*, based on the common spectral features. All investigated waxes are unsaturated, which is indicated by the aliphatic ν(C=C) stretching band around 1626 cm^−1^, the very weak ν(=CH) band around 1268 cm^−1^ and some stretching bands arising from aromatic compounds, e.g., the ν(CCH) band near 1602 cm^−1^ and the ρ(CH_2_) bending band around 886 cm^−1^. The high spectral similarity of the waxes of follicles sourced from different sites suggests that they do not differ in their composition. Waxes generally show distinctive spectral features in the C—H stretching region from 2750–3050 cm^−1^ when compared to resins, e.g., pine resin (Figure 5F).^20^ Typical resin features are absent in all of the spectra of *B. attenuata*. While we cannot exclude the presence of resins completely (despite the absence of resin features), if present, their concentration must be very low.

To test whether the melting temperature of the waxes differs between the follicles collected across the clinal range, we selected cones from sites 1 (northernmost), 5 (southernmost), and 3 (mid), and determined the phase transitions of the waxes in situ with combined heating and Raman spectroscopy. The conformational changes in the CH_2_ stretching region (2750–3050 cm^−1^) upon heating are demonstrated in the stack plot (Figure 5G) for the follicle waxes from site 1; and the changes of the intensity ratio *R* are plotted as a function of temperature (Figure 5H) for the three sampling sites 5, 3, and 1. As temperature increases, the lipid molecules undergo conformational changes from the ordered to disordered state, causing a shift of the maxima positions of the methylene symmetric and asymmetric bands near 2843 and 2877 cm^−1^ toward higher wavenumbers (2–3 and 8–12 cm^−1^, respectively), along with band broadening and the inversion of their relative band intensity. Due to the decline in overall band intensity with increasing temperature, noise also increased in the spectra, which in turn affects the broader distribution range of *R* at higher temperature values in Figure 5F.

The values of *R* display their maximum change from about 0.73–1.09 for all three sites during wax melting in the range of ≈45–51 °C.

### 2.5 Crack Formation in the Fracture Zone

**Figure 6:**
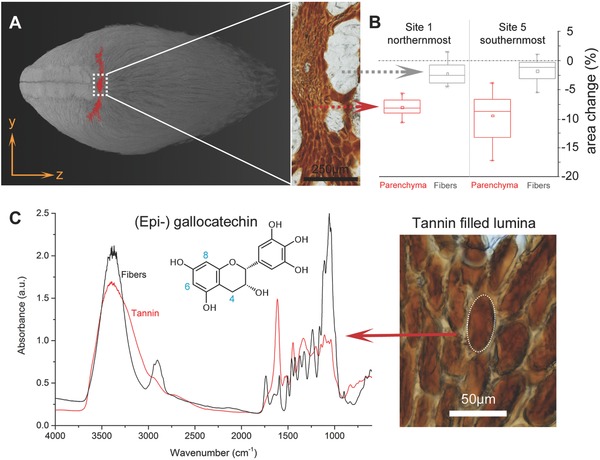
Thermal expansion and chemical composition of the fracture zone. A) Location highlighted in red in a µCT image and an unstained light micrograph (zoom) of the same region with fibrous and parenchymatic tissue. B) Dimensional changes of tissue sections exposed 80 °C relative to RT (parenchyma, red; fibers, gray) for the two extreme sites. C) In situ FTIR spectra obtained from the tannin filled cell lumina (inset) and fibers close to the FZ. The tannins show clear spectral bands of polymerized (epi‐)gallocatechin.

During initial opening, the valves separate along the junction zone (Figure 4A). This process is accompanied by the formation of a lateral crack, whose development and propagation are facilitated by the lack of endocarp tissue, a reduced valve thickness (Figure 3E), and the absence of mechanically strong fiber bundles (Figure 3C, white arrow).^22^ Light microscopy showed that the fracture zone contains a high amount of parenchymatic tissue filled with tannins (Figure 4D). The shape of the parenchyma corresponds well with the geometry of the developing crack (**Figure** **6**A). Dimensional changes of the two tissue types were measured upon heating of 20 µm thin sections to test whether crack initiation may be caused or facilitated by different thermal expansions of parenchymatic and fibrous tissue. The results confirm a differential thermal expansion since the area of the parenchymatic tissue shrank considerably more than the surrounding fibrous tissue (Figure 6B). However, no statistical differences between the two extreme sampling sites could be found. The parenchymatic tissue of the fracture zone is characterized by thin cell walls that enclose large amounts of condensed tannins (red/brownish appearance, Figure 6C). The incorporation of tannins in parenchymatic cell lumina is not limited to the FZ, but can also be found in other parts of the valves, especially in the mesocarp, where the fiber bundles are surrounded by a network of parenchymatic cells filled with condensed tannins.

The Fourier transform infrared (FTIR) spectra (Figure 6C) of follicle tannins from the two extreme sites (1 and 5) investigated show that they can be classified as condensed tannins, which is due to the absence of a strong carbonyl stretching band between 1750 and 1740 cm^−1^ that is typically only found in hydrolysable tannins.^23^ Therefore, the tannins are composed of the flavan unit as a basic structure. Based on the presence of weak bands at 1720 and 1145 cm^−1^, these units appear to be polymerized rather than associated as dimers.^24^, ^25^ The peak around 1613 cm^−1^ is typically assigned to stretching motions of the C—C bonds in the aromatic rings;^23^ with its relative intensity being influenced by the number of C4‐C8 interflavonoid linkages in polymeric structures.^26^ The relative intensity of this peak did not change after sample heating (Figure S1, Supporting Information); therefore, no further polymerization could be induced by heating. More structural information based on the spectral features can be derived from the region 1535–1520 cm^−1^. While flavonoids of the gallocatechin‐type display two peaks, simple catechin‐based compounds are characterized by a sharp singlet.^25^ A broadened band is expected for mixtures of both compounds. Due to the presence of a clear doublet in this region (Figure 6C), the detected flavonoids appear to be dominated by units of the (epi‐)gallocatechin‐type. This interpretation is supported by the disappearance of the double peak in heated samples with a simultaneous appearance of two shoulders in the region 1800–1700 cm^−1^ (Figure S1, Supporting Information), indicating that heating induced an oxidation of the hydroxyl groups, resulting in the formation of quinones.^27^ This process is also observable on the band centered around 1202 cm^−1^, previously assigned to stretching vibrations of the aromatic C—OH groups, which shows a decreased band intensity (relative to the aromatic ring stretching band near 1430 cm^−1^) with decreasing proportions of gallocatechin units in polymeric flavanol mixtures.^25^

### 2.6 Follicle Geometry Along the Environmental Gradient

**Figure 7:**
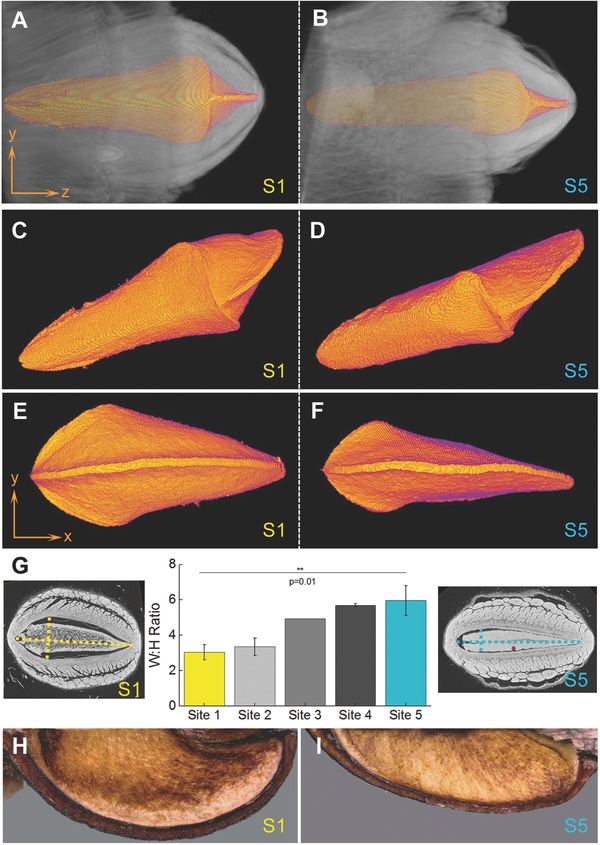
µCT‐based 3D geometry analysis of *B. attenuata* follicles. A,B) Longitudinal views of the follicle valves (gray) with the inner valve surface (orange, obtained via segmentation) for site 1 and site 5. C,D) Lateral and E,F) front views of the segmented inner valve surface for the two extreme sites revealing strong geometrical differences; especially the curvature in the tip region. G) W:H ratio (±SD) calculated from defined transversal follicle slices for all five sites. µCT images for S1 and S5 shown respectively on the left and right. H,I) Digital microscopy images (top view) of valves in the tip region, showing different curvatures.

A geometrical comparison of the asymmetric follicles of *B. attenuata* from the north (S1) and south (S5) populations is shown in **Figure** **7**. Segmented internal follicle volumes (including air, separator, and seeds; see Figure S2, Supporting Information) are displayed in both longitudinal (Figure 7A–D) and transverse direction (Figure 7E,F). Despite the asymmetry, it is clearly recognizable that the follicle valves generally exhibit a biaxially curved geometry (Figure 2A,B). By comparing the inner valve geometries of the northern‐ and southernmost samples (segmented colored volume in Figure 7A–F), pronounced geometric differences become visible (Figure 7H,I). Due to the irregular and complex valve geometry, we use the aspect ratio (width_max_:height_max_) of the inner valve surface in defined transverse follicle planes (Figure 7G and Figure S2, Supporting Information) for a quantitative comparison. The main geometric differences are reflected in strong curvature changes: at the driest site (S1, north), the valves are strongly curved (small W:H ratio) and flatten gradually toward the southern sampling sites (increasing W:H ratios in Figure 7G). Due to a higher sample number for sites 1 (*n* = 5) and 5 (*n* = 6), the data of these two sites was statistically analyzed; showing that the aspect ratios in follicles from the two extreme sites differ significantly (*p* = 0.01). From the X‐ray diffraction patterns (Figure 3A) and the work by Wardrop,^12^ it is already known that the endo‐ and mesocarp form a hygroscopic bilayer. By assuming a similar tissue composition, the described increase in the follicle curvature leads to an increase in its overall flexural rigidity—solely based on geometric stiffening^28^—as sampling sites progress north and opening temperatures increase. The aspect ratios show a strong negative correlation (Adj. *R* ^2^ = 0.980) with the follicle opening temperature (T50: *T* at 50% opening) for all investigated five sites (Figure S3, Supporting Information).

### 2.7 Differentiation between Temperature and Humidity Effects on Initial Opening

**Figure 8:**
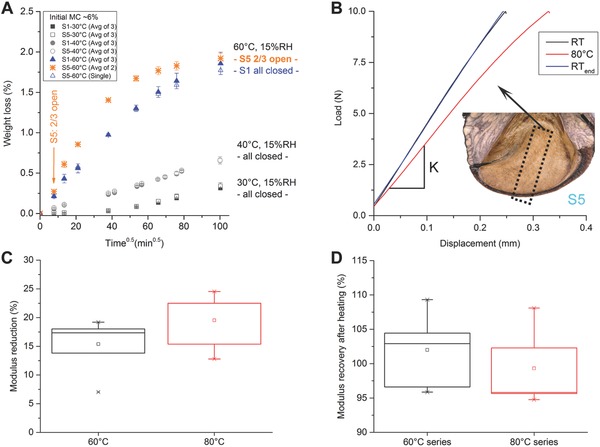
Humidity and temperature effects. A) Drying curves as a function of the square root of time for isolated follicles from the two extreme sites stored at different conditions with opening indicated by the arrow. B) Load–displacement curves for one sample experiencing three cycles of bending (RT–80 °C –RT_end_); revealing a temperature dependence of the elastic modulus in the endocarp (slope K changes). Inset: approximate sample location of the strips used for testing. C) Box plots representing the reduction of the elastic modulus of endocarp strips tested at 60 and 80 °C (*n* = 7 and *n* = 5, all for site 5 and relative to the values at RT ≈22 °C). D) Modulus recovery for both temperature series (comparing the values of RT vs. RT_end_).

Individual closed follicles from the two extreme sites do not show differences in the drying rates when exposed to the same conditions (**Figure** **8**A), indicating that their capacity for gas exchange is similar. Due to a greater water vapor saturation deficit (at constant RH), drying rates increase with increasing temperature. Opening, however, leads to increased moisture loss rates (Figure 8A, orange symbols vs. blue triangles), which can be explained by the sudden increase in surface area for potential gas exchange after opening. Surprisingly, the relative change in follicle moisture content after initial opening amounts only ≈0.25% for the two follicles from site 5 that opened within 30 min when exposed to 60 °C/15% RH (arrow in Figure 8A for *t* = 7.75 min^0.5^), whereas the third follicle from site 5 and all follicles from site 1 still remained closed even after 1 week; corresponding to ≈1.75%–2% weight loss of initially 6% moisture content. These observations clearly indicate that the initial opening step of the follicles is not triggered by a reduction in follicle moisture content, because all site 5 follicles that were exposed to 40 °C (and even one at 60 °C) remained closed at *t* = 100 min^0.5^ despite a greater weight loss compared to the ones opening at 60 °C at *t* = 7.75 min^0.5^.

Therefore, the key to follicle opening must be related to temperature. Based on the assumption that higher temperatures might influence the mechanical properties of the follicle tissue, we investigated the elastic modulus of longitudinal endocarp strips (Figure 8B, image) by bending experiments at different temperatures, since this layer acts as a resistance layer against bending along the longitudinal follicle axis. When subjected to loading (Figure 8B), the samples show a clear decrease in the elastic modulus at higher temperatures when compared to room temperature (Figure 8C). Once the samples were cooled down to RT, a final loading cycle was applied showing that the initial modulus was restored (Figure 8D). The temperature‐induced reduction of the modulus appears to be stronger at higher temperatures (Figure 8C). However, due to the scattering of the data, the difference between 60 and 80 °C is not statistically significant at the 0.05 level.

## 3 Discussion and Conclusions

In *B. attenuata*, the variation in the degree of serotiny has been linked to environmental parameters, with serotiny becoming more pronounced as annual rainfall decreases and mean temperature increases, and as the vegetation becomes more fire prone.^15^ For the first time, we show that increasing serotiny within a species is accompanied by an increase in the temperature required to initiate follicle opening (Figure 1C). The transition from weak to strong serotiny along the selected climatic gradient of our study is clearly reflected in a ≈20 °C shift in follicle opening temperatures (Figure 1 and Table S1, Supporting Information). Since the opening temperatures at the three northernmost sites exceed the highest daily air temperature recorded in January (maximum of ≈47 °C in the shade; Table S2, Supporting Information), follicle opening becomes more dependent on fire as the habitats become drier and hotter. However, these opening temperatures are much lower than previously reported (350–500 °C) for strongly serotinous *B. attenuata* plants collected from close to our most northern study site.^29^ While this earlier study employed a short duration of heat (2 min) to simulate the passage of a fire, we incrementally applied heat treatments over 24 h periods (Figure 1C). This approach enabled us to more precisely characterize opening temperatures, and to show that much lower temperatures can initiate follicle opening; but it is not a reproduction of environmental conditions that might be experienced in the natural ecosystem. We also show that drying under cool conditions (15 °C and 15% RH) is not sufficient to cause opening in the majority of follicles; a finding consistent with previous observations.^30^

Previous work has proposed that the melting temperature of resins in the junction zone might be correlated with the follicle opening temperature in some *Banksia* species.^12^ However, our study shows that waxes (cutin) are a predominant compound in the junction zone—a finding not reported elsewhere. These waxes are clearly not responsible for the variations in follicle opening, because their experimentally determined melting temperatures do not vary among sampling sites, unlike the opening temperatures. Further, the waxes are in the liquid state at around 50 °C, but follicles from the northern, drier sites (e.g., sites 1–3) still remain closed. Rather than an adhesive bonding agent, we propose that the role of the wax is probably more related to the frequently assigned functions of waxes in fruit and leaf structures; such as a barrier against water loss,^31^ an antiadhesive film against wetting, insects and microorganisms,^32^, ^33^ and a supporting filler material.^34^ Due to their relatively low melting temperature, we propose that these waxes could serve as a temperature‐activated filling material for microfissures in the junction zone. Such a mechanism could engender long‐term integrity and functionality of the follicle by sealing the seeds from harsh external conditions and providing an insulated environment conducive to seed protection and longevity in the canopy.

From the presence of large amounts of condensed tannins, we conclude that the follicle tissue has a high antioxidant capacity^35^ and potentially enhanced interactions with water molecules. A single phenol unit has amphiphilic properties; with the hydrophilic character originating from the occurrence of a free hydroxyl group, which can interact with water molecules by hydrogen bonding and dipole–dipole interactions.^27^ We expect that a high overall availability of polymerized flavan‐3‐ol structures with free hydroxyl groups, such as the prodelphinidins, might enable increased water retention by intra‐ and intermolecular sorption, because of the low binding affinities of epigallocatechin for other flavanols of the nongallate‐type in an aqueous environment.^24^ A high antioxidative capacity, together with an increased water retention capacity of the condensed tannins, might contribute to the structural integrity and dimensional stability of the follicles for long time periods in the absence of heat/fire. Condensed tannins are ubiquitous in higher plants and have important protective functions, including protection against pathogens and animal herbivores, but also protection against solar radiation, free radicals, and oxidants.^36^ In the context of fire‐resistant tree barks, condensed tannins have been mentioned to play a key role by being oxidized into large, planar graphite, and carbon molecules with heat insulating properties.^37^

The key to opening of *B. attenuata* follicles lies in the arrangement of the valve tissue on multiple length scales: based on the ultrastructural organization (Figure 3), pronounced longitudinal shrinkage of the mesocarp fibers is expected upon drying due to the high cellulose MFA, whereas the endocarp layer should be dimensionally more stable along the fiber axis as a result of the low cellulose MFA.^4^, ^7^, ^8^ As already mentioned, this has a tendency to induce bending of the bilayer formed by the meso‐ and endocarp, which is prevented by the integrity of the follicle, thus generating considerable residual stresses. The ability to resist these residual stresses is then determined on the macroscopic level by the valve geometry—namely, the curvature in the transverse and longitudinal follicle direction. It has been shown for thin sheets that by introducing a curvature in just one axis, a stiffening effect toward out‐of‐plane bending is achieved.^38^ For biaxially curved components like the follicles (ellipsoidal shells), the bending stiffness is even more influenced by curvature; with higher forces being required to achieve out‐of‐plane deformations in more strongly curved surfaces.^28^ In our system, similar relations apply due to the different W:H ratios identified in follicles from different climatic regions; implying that the follicles from the driest, most fire‐prone northern site have the highest flexural rigidity due to the strongly curved valves (Figure 7). As a consequence of the changing valve geometry, and hence flexural rigidity, the dimensional stability and opening temperatures of the follicles gradually increase toward the northern end of the gradient. Upon exposure to higher temperatures, the elastic modulus of the endocarp decreases (Figure 8); allowing the residual stresses in the fibers to be released (at least partially). Even though the modulus reduction from 60 to 80 °C is not statistically significant, we conclude from the observation that all follicles open at 80 °C within a relatively short time that a mean reduction of the endocarp elastic modulus by ≈22% seems to be sufficient to release residual stresses and, thus, trigger follicle opening.

We attribute the decreasing elastic modulus of the endocarp to a thermal softening of the lignin contained in the tissue, because of the relatively low glass transition temperature reported for lignin in the presence of residual water (*T*_g_ = 80–100 °C for MC = 5%–10%).^39^ As the valves deform, the structure in the exocarp and the area of the FZ are disrupted irreversibly, which enhances diffusion across the follicle–atmosphere interface and thereby stimulates further moisture driven swelling and shrinkage movements of the 3D bilayer.

These findings may add new ideas to the field of bioinspired engineering, where the concepts of bilayers and other programmed microstructures undergoing stimuli‐responsive 3D shape transformations are currently being widely explored; already revealing promising technical applications.^40^, ^41^, ^42^, ^43^, ^44^ In general, bending rigidity is based on two contributions, which are the geometry of the device and the elastic properties of the material it consists of. In *Banksia* follicles, a temperature‐induced softening, most likely due to a glass transition of lignin, facilitates bending of the valves, which is critical for the opening of the follicle. Since the elastic properties of the tissue do not depend on the collection site, changes in geometry of the bilayer are used to modulate the actual opening temperature.

From an ecological point of view, the previously stated follicle properties are particularly beneficial for seed protection in plants of the northern sites, because their follicles are likely to experience higher temperatures during bushfires due to the reduced height of the plants in these populations relative to the plants in more southern sites. As climate and follicle opening behavior of *B. attenuata* change along the gradient, so do vegetation structure, plant architecture, fire regimes, and fire exposure of *Banksia* cones. In the southern part of our gradient *B. attenuata* is a 6–8 m tall, canopy‐resprouting tree, while in the north it is typically a 1.5–2.5 m, multistemmed, basally resprouting shrub. This varying plant stature reflects varying structure of the vegetation; *B. attenuata* typically contributes to, or dominates, the canopy of a low woodland (tree form), or a species rich shrubland (shrub form).^45^ In the northern shrublands, fires typically burn all vegetation strata, including the canopy of *Banksia* plants; whereas in the woodlands, fires may burn understory strata alone, or the lower and canopy layers together. Data on the relative frequency of crown fires in these two systems is lacking, but it is likely that *Banksia* woodland crowns, where the cones are held, burn at a much lower frequency than those of shrublands. Thus, in woodlands, lower follicle opening temperatures could be beneficial for several reasons: (i) temperatures experienced by canopy‐stored cones from ground fires are likely to be lower, requiring a higher stimuli responsiveness; (ii) longer periods between fire experienced in the woodland canopy may mean that there is a lower likelihood that canopy stored seeds will encounter fire in their lifetime, and storage awaiting a fire trigger is not as effective a strategy, and; (iii) with relatively higher rainfall, germination of spontaneously released seeds may be more likely to lead to successful interfire seedling establishment and increasing long‐term survival.^15^ For the drier, more fire‐prone regions, we speculate that the specific opening temperatures prevent early seed release in these areas with a small likelihood of successful germination and establishment between fires.

## 4 Experimental Section

*Material Collection*: Mature infructescences of *B. attenuata* R. Br. were collected in May/June 2014 from five sites between Perth and Geraldton, in Western Australia which had not been burnt in the last ten years (Figure 1B and Table S2, Supporting Information). A second collection from sites 1 and 5 occurred in September/October 2016. With decreasing geographic latitude along this 350 km gradient, mean annual rainfall decreases and mean maximum air temperature in summer increases (Table S2, Supporting Information). At each site, five to ten randomly chosen, healthy individuals bearing closed, intact follicles at the age of one to five years were selected to take sample material. All collected infructescences were stored in cotton/paper bags at room temperature until further analysis.

*Opening Experiments*: A total of 50 collected infructescences (10/site) were stored at constant conditions (15% RH; 15 °C) for approximately two weeks to balance local humidity differences. Five infructescences of each site were then put in an oven (40 °C, 24 h) and temperature stepwise increased (+10 °C every 24 h up to 103 °C). For monitoring of the actual conditions in the oven a measurement module (NI cDAQ‐9171/NI9211) consisting of one combined humidity and temperature sensor (SHT75, Sensirion) and two thermo elements (K406‐484, TC Direct) together with an in‐house developed multitemperature control software were used. Every 24 h, after cooling of the samples in an airtight container with silica gel (10 min), samples were weighed and the number of open follicles on each infructescence was counted. The same procedure (without heating) was applied to the five reference infructescences of each site, stored at 15 °C and 15% RH (cold drying). In order to confirm that the reference follicles were intact and able to open, they were put in an oven (103 °C, 24 h) after the experiment. Follicle opening was defined as the point where a visible separation of the junction zone occurred (between the two woody valves). The data points were fitted with a nonweighted Boltzmann fit (fixed curve slope of d*x* ≥ 1) in OriginPro (version 9.1, OriginLab) in order to determine the temperature values for 50% follicle opening.

*Sample Preparation*: A band saw was used to isolate follicles from the infructescences. Thin sections for histochemical staining, Raman spectroscopy (20 µm, transversal cuts), FTIR spectroscopy (6 µm, longitudinal) were obtained with a rotating microtome (RM2255, Leica).

*Histochemical Staining*: In order to test the presence of lipids, follicle sections were stained with Sudan IV by immersing the tissue in a few drops of Sudan IV (1 g) in ethanol (50 mL, 96%) and glycerol (2.5 mL), followed by short heating and rinsing with distilled water.

*FTIR Spectroscopy*: Longitudinal sections (6 µm) of the FZ of follicles from site 5 and site 1 were attached to a plastic sample holder and analyzed in transmission mode in the range of 4000–600 cm^−1^ with an FTIR Microscope (Hyperion 2000, Bruker) connected to an mercury cadmium telluride (MCT) detector. Absorbance spectra were acquired from air‐dried/nonheated and heated sample sections (100 °C for 5 min on a heating plate) with a spectral resolution of 2 cm^−1^ with each single spectrum being an average of 32 scans. As far as possible, only the cell contents of several big FZ cells were measured for tannin identification, without the surrounding cell walls.

*Confocal Raman Spectroscopy*: Imaging was performed on wet transversal sections (20 µm) with a confocal Raman microscope (alpha300RA, WITec) optimized for 785 nm laser (Xtra‐PS, TOPTICA Photonics) wavelength by having a blazed grating (600 g mm^−1^, UHTS spectrometer, WITec) and a deep‐depletion camera (ANDOR, DU401A BR‐DD) coupled via fiber optics to the microscope. The laser light was focused through a 100× oil immersion objective (NA 1.4, Zeiss). The scan parameters were set to an integration time of 0.8 s per spectrum, 90 points per line, and 90 lines per image (30 × 30 µm). The collected hyperspectral dataset was analyzed via Non‐Negative Matrix Factorization (NMF) in Project FOUR (WITec) with three basis spectra and 10 000 iteration steps after applying a cosmic ray removal and baseline correction. The characterization of the chemical composition and the phase transition of the waxes in the junction zone during gradual heating were performed with a confocal Raman Microscope (CRM200, WITec) in combination with a piezo‐scanner (P‐500, Physik Instrumente) and a diode pumped 785 nm NIR laser excitation source (Toptica Photonics). The laser beam (1 µm spot size) was focused down on the tissue sections through a 20× microscope objective (NA 0.40, Nikon). Sections from three different follicles of each of the sites 1, 3, and 5 were measured in the wet state with a self‐developed experimental setup: the sections were clamped on top of an insulated copper pot filled with distilled water, which was in contact with the sample. The pot/water was heated with a heating stage (FTIR‐600, Linkam) and the temperature monitored with two thermo elements (K406‐484, TC Direct). Single spectra were acquired at 25–56 °C with an integration time of 5 s after 2 min of equilibration with a grating of 300 g mm^−1^ using a charge‐coupled device (CCD) camera (PI‐MAX, Princeton Instruments) and a spectrograph (Acton, Princeton Instruments) with a spectral resolution of ≈6 cm^−1^. The background of the spectra was subtracted manually in WiTecProject (version 2.08, WITec) after the acquisition with ScanCtrlSpectroscopyPlus (version 1.38, WITec).

The quantitative characterization of the phase transitions was performed by calculating the intensity ratio *R* of the signal intensity (only if CCD cts > 80) for the CH_2_ stretching band at 2885 cm^−1^ relative to the one at 2880 cm^−1^ as a function of temperature; as suggested by Lewis and McElhaney.^21^ The melting temperature of the wax was determined from the inflection point of a sigmoidal, nonweighted concatenated Boltzmann fit on the data set in OriginPro (version 9.1, OriginLab).

Previous to the experiment, this methodology was tested successfully to determine the phase transition (75.8 ± 1.4 °C with Adj. *R* ^2^ = 0.88) in No. 1 Sigma‐Aldrich yellow Carnauba wax heated on top of a quartz object slide. Carnauba wax is a plant‐based wax, produced by *Copernicia prunifera*, which is used as a reference. The viscous resin from *Pinus banksiana* was obtained from a tree in the Forest Botanical Garden in Tharandt (Technical University Dresden). The cutin reference spectrum was recorded, described, and kindly provided by P. Mateu et al.^18^

*µCT*: Closed *B. attenuata* follicles (three from each site collected in 2014 and three additional ones from sites 5 and 1 from the year 2016) were scanned with a Phoenix v|tome|x s laboratory X‐ray computed tomograph.^46^ Scanning parameters were set to 80 keV tube voltage and 320 µA current. A single scan consists of 1200 2D radiographs (900 for fast scans during heating) with variable voxel sizes (13.5–19 µm) due to different sample sizes and a regular rotation step size over 360°. Heating occurred via two parabolic mirrors that were mounted next to the sample and perpendicular to the path of the X‐rays. The radiographs were reconstructed in phoenix datos|x (Version 2.1, GE Sensing & Inspection Technologies) using the conventional 2D filtered back projection algorithm.^47^ Segmentation and further analysis of regions of interest was performed by using the 3D analysis software Amira 6 (ZIB Edition, FEI). The orientation of the section plane is illustrated in Figure S2 (Supporting Information). The selected 2D slices were analyzed in Fiji (ImageJ 2.0, National Institutes of Health).

*Wide Angle X‐Ray Diffraction (WAXD)*: WAXD measurements were performed at the µspot beamline at BESSY II using synchrotron radiation with an energy of 15 keV and a beam size of 30 µm. 20 µm thin longitudinal sections were fixed on aluminum frames and then mounted perpendicular to the incident monochromatic beam. On three samples five line scans with a step size of 100 µm, containing all pericarp layers (endo‐, meso‐, and exocarp) were performed. The exposure time was 20 s and the scattering patterns were detected with a 2D CCD detector with 3072 × 3072 pixels and a pixel size of 73.25 µm (MarMosaic 225, Evanston).

*Mechanical Testing*: Endocarp strips (*L* = 1–2 cm × *W* = 3–5 mm × *T* = 1–2 mm) were isolated with a hand saw from site 5 follicles and then polished on the cut side to a uniform thickness and smooth surface with sandpaper. A three‐point‐bending test was performed with a 2.5 kN mechanical testing machine (zwicki, Zwick Roell), equipped with a 1 kN load cell. The strains were measured by cross‐head travel. An infrared spot (Optron, 150 W/50 mm focal point distance) was used for heating. The samples were loaded in transverse direction (endocarp surface up) with a distance of 8 mm of the supporting pins in three testing cycles (RT–60 °C/80 °C–RT_end_) with a maximum force of 10 N, a preload of 0.2 N, and a test speed of 0.05 mm s^−1^. Samples that showed plastic deformation or failure in the first loading cycle (at RT) or a modulus recovery different to 90%–110% (at RT_end_) were excluded from further testing cycles and analysis. The elastic modulus *E* was calculated as a product of the sample geometry (length *L*; thickness *d*, and width *b*) and the slope of the linear regime in the load *F* versus displacement *x* curve according to Equation (1)

(1) $$E=\frac{L^{3}}{4bd^{3}}\frac{\Delta F}{\Delta x}$$

*Thermal Expansion*: Thermal expansion of the fracture zone parenchyma and the surrounding fibrous tissue of follicles from site 1 and site 5 were investigated on 20 µm thin longitudinal sections containing both fracture zone parenchyma and fibrous tissue (Figure 6). The sections were trimmed to rectangular shaped samples and afterward transferred to an electrically conductive indium tin oxide (ITO) coated glass slide. A thermo element (K406‐484, TC Direct) was in contact with the glass slide to control and monitor the temperature of the heat which was generated by applying a voltage to the ITO layer and the resultant current flow. The device was placed under a transmission light microscope and the samples were heated up from room temperature to at least 80 °C. Every 5 °C an image was taken. The areas of both tissues of each slice were determined using defined thresholds in Fiji or manually segmented (depending on quality of the micrographs).

*Statistical Analysis*: All mathematical and statistical analysis was performed in OriginPro (Version 9.1, OriginLab). The Mann–Whitney test was selected for comparing the aspect ratios between sites and also for comparing the mechanical testing data using significance levels of 0.05 (*), 0.01 (**), and 0.001 (***).

## Conflict of Interest

The authors declare no conflict of interest.

## Supporting information

Supplementary

## References

[1] J. Fromm , S. Lautner , Plant, Cell Environ. 2007, 30, 249. 17263772 10.1111/j.1365-3040.2006.01614.x

[2] Y. Forterre , J. M. Skotheim , J. Dumais , L. Mahadevan , Nature 2005, 433, 421. 15674293 10.1038/nature03185

[3] R. Elbaum , Y. Abraham , Plant Sci. 2014, 223, 124. 24767122 10.1016/j.plantsci.2014.03.014

[4] S. Armon , E. Efrati , R. Kupferman , E. Sharon , Science 2011, 333, 1726. 21940888 10.1126/science.1203874

[5] R. Elbaum , L. Zaltzman , I. Burgert , P. Fratzl , Science 2007, 316, 884. 17495170 10.1126/science.1140097

[6] I. Burgert , P. Fratzl , Integr. Comp. Biol. 2009, 49, 69. 21669847 10.1093/icb/icp026

[7] R. Allen , A. B. Wardrop , Aust. J. Bot. 1964, 12, 125.

[8] J. Dawson , J. F. V. Vincent , A. M. Rocca , Nature 1997, 390, 668.

[9] M. J. Harrington , K. Razghandi , F. Ditsch , L. Guiducci , M. Rueggeberg , J. W. C. Dunlop , P. Fratzl , C. Neinhuis , I. Burgert , Nat. Commun. 2011, 2, 337. 21654637 10.1038/ncomms1336

[10] T. H. He , B. B. Lamont , K. S. Downes , New Phytol. 2011, 191, 184. 21388378 10.1111/j.1469-8137.2011.03663.x

[11] B. B. Lamont , R. M. Cowling , Aust. J. Ecol. 1984, 9, 295.

[12] A. B. Wardrop , Aust. J. Bot. 1983, 31, 485.

[13] M. D. Crisp , L. G. Cook , Annu. Rev. Ecol. Evol. Syst. 2013, 44, 303.

[14] D. T. Bell , Bot. Rev. 2001, 67, 417.

[15] R. M. Cowling , B. B. Lamont , Aust. J. Ecol. 1985, 10, 345.

[16] B. B. Lamont , S. W. Connell , J. Biogeogr. 1996, 23, 295.

[17] A. S. George , Nuytsia: The Genus Banksia L.f. (Proteaceae), Western Australian Herbarium, Department of Agriculture, Perth, 1981.

[18] B. P. Mateu , M. T. Hauser , A. Heredia , N. Gierlinger , Front. Chem. 2016, 4, 10. 26973831 10.3389/fchem.2016.00010 PMC4770935

[19] H. G. M. Edwards , M. J. P. Falk , Spectrochim. Acta, Part A 1997, 53, 2685.

[20] H. G. M. Edwards , D. W. Farwell , L. Daffner , Spectrochim. Acta, Part A 1996, 52, 1639.

[21] R. N. A. H. Lewis , R. N. McElhaney , in Handbook of Vibrational Spectroscopy, (Eds.: Chalmers J. M., Griffiths P. R.), John Wiley & Sons, Ltd., Chichester 2006.

[22] L. J. Gibson , J. Biomech. 2005, 38, 377. 15652536 10.1016/j.jbiomech.2004.09.027

[23] A. Ricci , K. J. Olejar , G. P. Parpinello , P. A. Kilmartin , A. Versari , Appl. Spectrosc. Rev. 2015, 50, 407. 10.1366/15-07957 26647047

[24] T. Ujihara , N. Hayashi , J. Nat. Prod. 2016, 79, 66. 26720794 10.1021/acs.jnatprod.5b00658

[25] L. Y. Foo , Phytochemistry 1981, 20, 1397.

[26] S. Kim , H. J. Kim , J. Adhes. Sci. Technol. 2003, 17, 1369.

[27] S. Quideau , D. Deffieux , C. Douat‐Casassus , L. Pouysegu , Angew. Chem., Int. Ed. 2011, 50, 586. 10.1002/anie.201000044 21226137

[28] A. Lazarus , H. C. B. Florijn , P. M. Reis , Phys. Rev. Lett. 2012, 109, 144301. 23083245 10.1103/PhysRevLett.109.144301

[29] N. J. Enright , B. B. Lamont , Aust. J. Ecol. 1989, 14, 107.

[30] A. M. Gill , Aust. J. Bot. 1976, 24, 329.

[31] H. Bargel , K. Koch , Z. Cerman , C. Neinhuis , Funct. Plant Biol. 2006, 33, 893. 10.1071/FP06139 32689300

[32] C. Buschhaus , R. Jetter , J. Exp. Bot. 2011, 62, 841. 21193581 10.1093/jxb/erq366

[33] T. S. Wong , S. H. Kang , S. K. Y. Tang , E. J. Smythe , B. D. Hatton , A. Grinthal , J. Aizenberg , Nature 2011, 477, 443. 21938066 10.1038/nature10447

[34] P. D. Petracek , M. J. Bukovac , Plant Physiol. 1995, 109, 675. 12228622 10.1104/pp.109.2.675 PMC157635

[35] C. A. RiceEvans , N. J. Miller , G. Paganga , Free Radicals Biol. Med. 1996, 20, 933. 10.1016/0891-5849(95)02227-9 8743980

[36] W. Vermerris , R. L. Nicholson , Phenolic Compound Biochemistry, Springer, Dordrecht 2008.

[37] H. Tributsch , S. Fiechter , WIT Trans. Built Environ. 2008, 97, 43.

[38] V. Pini , J. J. Ruz , P. M. Kosaka , O. Malvar , M. Calleja , J. Tamayo , Sci. Rep. 2016, 6, 29627. 27403938 10.1038/srep29627 PMC4939595

[39] C. Wang , S. S. Kelley , R. A. Venditti , ChemSusChem 2016, 9, 770. 27059111 10.1002/cssc.201501531

[40] S. Felton , M. Tolley , E. Demaine , D. Rus , R. Wood , Science 2014, 345, 644. 25104380 10.1126/science.1252610

[41] Y. Q. Mao , Z. Ding , C. Yuan , S. G. Ai , M. Isakov , J. T. Wu , T. J. Wang , M. L. Dunn , H. J. Qi , Sci. Rep. 2016, 6, 24761. 27109063 10.1038/srep24761 PMC4842966

[42] M. Ruggeberg , I. Burgert , PLoS One 2015, 10, 1. 10.1371/journal.pone.0120718 PMC4383548 25835386

[43] A. R. Studart , R. M. Erb , Soft Matter 2014, 10, 1284. 24651249 10.1039/c3sm51883c

[44] Z. Zhao , J. Wu , X. Mu , H. Chen , H. J. Qi , D. Fang , Sci. Adv. 2017, 3, e1602326. 28508038 10.1126/sciadv.1602326 PMC5409495

[45] B. P. Miller , K. W. Dixon , in Plant Life on the Sandplains in Southwest Australia, a Global Biodiversity Hotspot (Ed: Lambers H.), UWA Press, Perth 2014.

[46] J. Y. Buffiere , E. Maire , J. Adrien , J. P. Masse , E. Boller , Exp. Mech. 2010, 50, 289.

[47] L. A. Feldkamp , L. C. Davis , J. W. Kress , J. Opt. Soc. Am. A 1984, 1, 612.

