# Supplementary material for: Climate‐Dependent Heat‐Triggered Opening Mechanism of Banksia Seed Pods
Source: Adv Sci (Weinh). 2017 Dec 13;5(1):1700572. doi: 10.1002/advs.201700572 (PMC5770687; doi:10.1002/advs.201700572)
Supplement: Supplementary file 1 — Supplementary [file ADVS-5-na-s001.pdf]

Copyright WILEY-VCH Verlag GmbH & Co. KGaA, 69469 Weinheim, Germany, 2017.

ADVANCED SCIENCE
Open Access

Supporting Information

for *Adv. Sci.,* DOI: 10.1002/advs.201700572

Climate-Dependent Heat-Triggered Opening Mechanism of *Banksia* Seed Pods

Jessica C. Huss, Vanessa Schoeppler, David J. Merritt, Christine Best, Eric Maire, Jérôme Adrien, Oliver Spaeker, Nils Janssen, Johannes Gladisch, Notburga Gierlinger, Ben P. Miller, Peter Fratzl, and Michaela Eder**

DOI: 10.1002/ ((advs.201700572))
Article type: Full Paper

Climate-Dependent Heat-Triggered Opening Mechanism of *Banksia* Seed Pods

Jessica C. Huss, Vanessa Schoeppler, David J. Merritt, Christine Best, Eric Maire, Jérôme Adrien, Oliver Spaeker, Nils Janssen, Johannes Gladisch, Notburga Gierlinger, Ben P. Miller, Peter Fratzl, Michaela Eder**

Supporting Information

Climate-Dependent Heat-Triggered Opening Mechanism of Banksia Seed Pods

Jessica C. Huss, Vanessa Schoeppler, David J. Merritt, Christine Best, Eric Maire, Jérôme Adrien, Oliver Spaeker, Nils Janssen, Johannes Gladisch, Notburga Gierlinger, Ben P. Miller, Peter Fratzl, Michaela Eder**

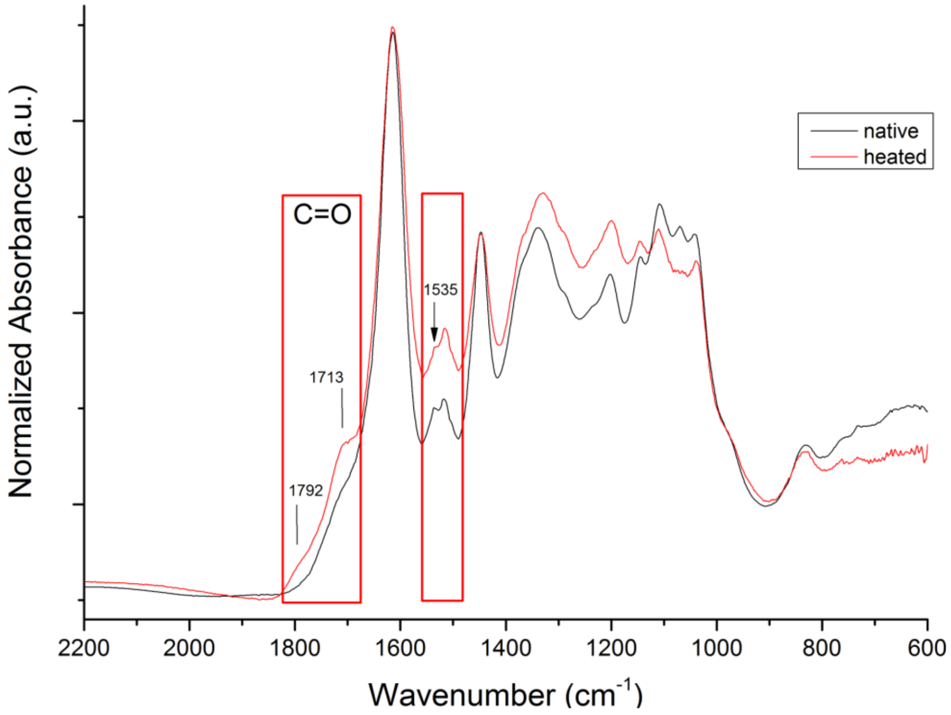

Figure S1. FT-IR average spectra (*in situ*) of condensed FZ tannins of *B. attenuata* follicles in the native and heated state. Averages of 6 spectra each. Highlighted areas show features that accompany oxidation/ the formation of quinones. Heated samples had dark brown coloured tannins (oxidized) under the light microscope, whereas the air-dried samples were red.

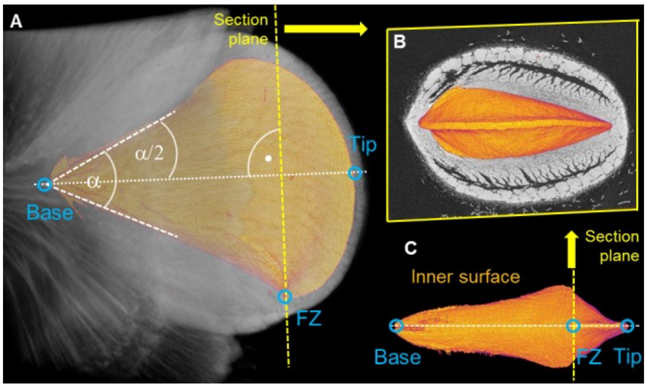

Figure S2. Axis orientation for the 3D geometry analysis of *B. attenuata* follicles. (A) top view of a follicle (grey), its inner surface (colored) and the orientation planes (white) used for defining the section plane (yellow). (B) front view of the 2D plane of interest and the inner 3D surface. (C) Lateral view of the inner surface indicating the section plane.

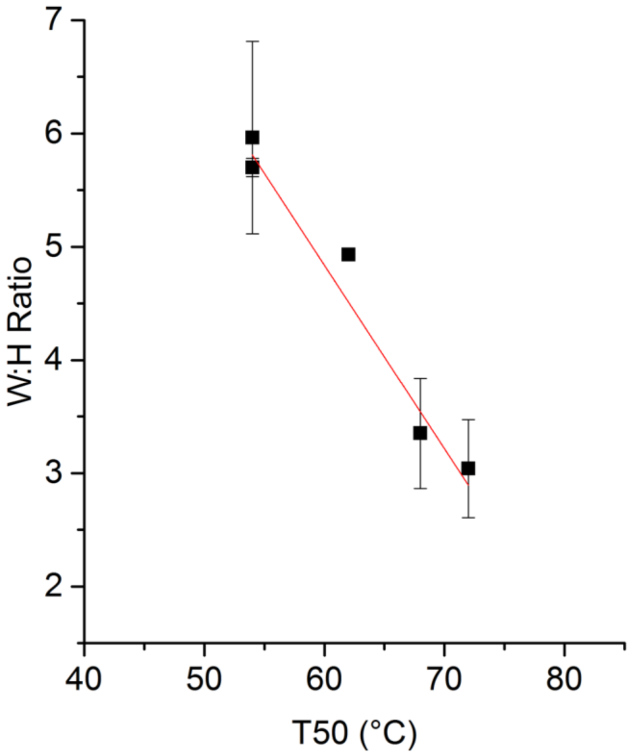

Figure S3. Negative linear correlation (Adj. R^2=0.980) between the width:height ratio and the opening temperature at 50% open follicles (T50) plotted for all five sites. The opening temperature increases with a decreasing W:H ratio. Low W:H ratios correspond to high principle valve curvatures; a circular cylinder cross section has a W:H ratio of 1 (equals the radius).

Table S1. Sequence of temperature treatment of follicles until opening

	Treatment conditions		Proportion of open follicles (%)				
duration	Temperature T (°C)	Relative Humidity RH (%)	Site 1 (*n*=78)	Site 2 (*n*=73)	Site 3 (*n*=56)	Site 4 (*n*=63)	Site 5 (*n*=39)
n.a. a)	n.a.	n.a.	0	10	23	51	44
2 weeks b)	15	15	0	11	23	51	44
24 hours c)	37	15	0	11	23	51	44
24 hours	47	12	0	11	23	52	49
24 hours	57	7	0	12	23	84	85
24 hours	67	5	1	34	100	92	100
24 hours	77	2	86	100	100	92	100
24 hours	87	0	89	100	100	92	100
24 hours	97	0	89 d)	100	100	92 d)	100

a) directly after collection; b) 2 weeks storage of all follicles under controlled conditions; c) subsequent treatments were sequentially imposed each for a 24 hour period; d) some follicles did not open, which could be due to fungal/ insect or other structural damage

Table S2. Environmental parameters at the sampling sites of *B. attenuata*

Site	Geographic position	Mean annual rainfall [a)] $P_{a,mean}$ (mm)	Mean max. air temperature in January [a)] $T_{mean,max,Jan}$(°C)	Highest daily max. air temperature in January [a)] $T_{max,Jan}$ (°C)	Plant Morphology
1	29.62080°S 115.21177°E and 29.62343°S 115.21430°E	469.1	36.4	47.3	Shrub
2	30.04438°S 115.32618°E	546.5	36.4	47.3	Shrub
3	30.57174°S 115.46120°E	600.1	34.7	46.9	Tree
4	31.18664°S 115.80159°E	687.1	n.a.	n.a.	Tree
5	31.69048°S 115.87981°E and 31.68662°S 115.88483°E	807.0	33.5	46.0	Tree

[a)] Data retrieved from the Bureau of Meteorology (Australian Government) at the closest weather station within a 30 kms range and a minimum data record of 15 years in between 1900 and 2014. Data can be obtained free of charge via http://www.bom.gov.au/climate/data/stations/
